# Marine constraints as philosophical opportunities: the Krogh principle and the benefits of philosophical engagement with the sea

**DOI:** 10.1007/s40656-025-00688-0

**Published:** 2025-09-11

**Authors:** Elis Jones, Vincent Cuypers

**Affiliations:** 1 Egenis Centre for the Study of Life Sciences, University of Exeter, Exeter, United Kingdom; 2 Konrad Lorenz Institute for Evolution and Cognition Research, Klosterneuburg, Austria; 3 Research Group Zoology: Biodiversity and Toxicology, Centre for Environmental Sciences, Hasselt University, Diepenbeek, Belgium; 4 Institute of Philosophy, KU Leuven, Leuven, Belgium

**Keywords:** Marine philosophy, Krogh principle, Meta-philosophy of science, Epistemic constraints, Marine science

## Abstract

This paper shows how cases drawn from the marine sciences can be particularly fruitful for philosophical reflection about the nature of science. We offer a meta-philosophical adaptation of a heuristic (the Krogh Principle) taken from comparative biology, drawing connections between a problem common to both biology and philosophy of science: how to apportion scarce attention between the bewildering array of potential study systems? And how to do so in a way which recognises the diversity of those study systems, but preserves the possibility of generalisation? The Krogh Principle offers a heuristic: choose cases where the phenomenon of interest is demonstrated in an extreme or unusual way, so as to make the phenomenon particularly accessible. We follow one particular sub-strategy, namely, the exploration of cases which are subject to strong environmental constraints, which we expect to be as fruitful in the choice of organisms as it is for scientific case studies. Marine sciences offer examples of substantial environmental constraints on scientific practice, and so present extreme and unusual examples from which philosophers can improve existing conceptual machinery to the benefit of both philosophers and scientists. In particular, we use examples from coral reef and deep-sea science to show how marine sciences can both reinforce and refine philosophical understanding of the role played by values in science. We conclude by suggesting that many other topics – in both philosophy and science - may also stand to benefit from philosophical engagement with environmentally-constrained or otherwise unusual case studies, in particular cases taken from the marine sciences.

## Introduction

In the beginning, there was physics. At least, in the beginning of philosophy of science, there was philosophy of physics. This was true to the extent that Ernst Mayr, a key figure in the development of modern philosophy of biology, argued that several volumes he owned claiming to be ‘philosophy of science’ should rather have been titled ‘philosophy of physics’. Coupled with this was the complaint, echoed by others, that generalisations about science drawing only on physics do not hold for much of biology (Mayr, 1969; Butterfield, 2016).

Since then, a great many ‘philosophies of’ have bloomed under the umbrella of philosophy of science. Increasing awareness that each scientific discipline has its own specific peculiarities and challenges has led philosophers of science to explore many new frontiers (Rouse, 2023). Much philosophical work is now being done on biology, the environmental sciences (Odenbaugh & Griffiths, 2022), medicine (Broadbent, 2019), earth sciences (Ohnesorge & Watkins, 2024), historical sciences (Currie & Turner, 2016), economics and other social sciences (Reiss, 2013; Risjord, 2022), chemistry (Weisberg et al., 2011), and many more topics.

However, the problem of generalising across disciplines and cases persists. The proliferation of ‘philosophies of’ various ‘special sciences’ means that much of philosophy of science de facto operates with a focus on the disunity, rather than the unity of science. The increased awareness of the diversity of scientific practices is, we believe, very much an improvement on trying to generalise from one science (such as physics) to all of the others, but it raises further challenges. Indeed, recognising that scientific activity is diverse, involving different methods, techniques, goals and norms (Dupré, 1993; Cartwright, 1999), threatens the ability of philosophers to reflect upon and generalise about it as a whole at all (Rouse, 2014; Currie, 2015; Potochnik, 2017; Schindler & Scholl, 2022). If science is as diverse as many now claim, can there still be such thing as generalised philosophy of science? Is it still meaningful to try to develop concepts and theories which stretch across different areas of science? How can this be done?

An important part of this challenge - raised by the recognition of the diversity of scientific practices - relates to the distribution of research attention in philosophy of science. If there is not one, but many sciences, which ones should be studied and when? The resources of philosophy of science as a discipline are limited and should be well-spent. Not all scientific activities can realistically be put under philosophical scrutiny. Selecting the right study systems, the most relevant areas of science, for philosophical analysis is a key part of ensuring a ‘well-ordered’ (Kitcher, 2001) and ‘well-rounded’ philosophy of science (Mayr, 1969, p. 202).

Under the umbrella of ‘science’ are a very large number of potentially interesting examples and case-studies, and one risks being indiscriminate about their actual relevance and usefulness (Currie, 2015). As a result, there have been worries about biases and unrepresentativeness in the use of case-studies in the underpinning of philosophical theories (Currie, 2015; Mizrahi, 2020; Schindler & Scholl, 2022). This calls for further reflection about what might guide choice of areas of investigation for philosophers of science.

In this paper we make two contributions. First, a meta-philosophical one: we offer a principle, adapted from comparative biology – the Krogh principle – for heuristically guiding the choice of study systems in philosophy of science in the wake of the problem of the diversity of science. In a nutshell, the Krogh principle in comparative biology suggests that study organisms can be picked because they display a phenomenon of interest in an extreme or unusual way (a marked difference from picking organisms because they display it in a typical or familiar way). One version of this argument focuses on the role of environmental constraint in producing such extreme or unusual displays. Such cases can allow for insights into the fundamental features of some phenomenon and how they relate to environmental constraints. In a similar way, we think that those areas of science that are somehow extreme cases relative to more quotidian or well-studied scientific activities are likely to be particularly fruitful sites for philosophical scrutiny.

Second, we build on this principle to show that the marine sciences constitute a particularly useful study system for the investigation of key philosophical topics. The marine sciences grapple with strong environmental constraints – the sea is inhospitable for scientists – and how marine scientists navigate the harsh environment in which they work can reveal much about how science works. Our focus here is on the interaction between both environmental and social factors, more specifically, how the values which are well-understood to guide scientific practice can be understood more thoroughly by considering how scientists operate in physically constrained environments.

In doing so, we want to go beyond the notion that marine science is simply a large area of work neglected by philosophers of science (this is true, but many areas of science are, for the simple reason of a paucity of philosophers), or that marine environments are particularly important for human survival and so worthy of philosophical attention (this is also true, but it might be argued that many other environments are too). Rather, we offer a meta-philosophically principled reason for expecting particularly large benefits to come from attending to the sea, alongside examples of these benefits as visible in a core topic of interest to philosophers. In turn, we hope it illustrates the value of the Krogh principle for the selection of study systems in philosophy of science, and points to a future for philosophy of science as a serendipitous patchwork of diverse case studies brought into fruitful dialogue with one another.

## Selecting study systems in philosophy of science: the Krogh principle

Drawing on contingent and context-specific areas of science in order to make generalisations about the nature of scientific activity might not seem like a sound strategy. As the old academic jibe goes, one might be at risk of drawing a line of best fit from a single data point. This holds at different levels: with regard to the broader area of science one focuses on - the particular ‘philosophy of’ one adheres to - but also to selection of case-studies within such a field.

Schindler and Scholl (2022) nonetheless defend the repeated use of specific well-studied cases in philosophy of science. They analogise, as we do here, between how biologists choose organisms to study and how philosophers choose areas of science to study. They argue that as with model organisms, case studies can be informative beyond their original contexts due to the similarities they share with other cases, in particular because scientific practices are shared across different scientific groups, stretching over space and time, so that understanding one case is likely to help with understanding others (just as model organisms may be taken to be scientifically useful due to their common ancestry, or similarity, with other organisms (Ankeny & Leonelli, 2020).

There are further lessons to be drawn from reflections on the targeting of scarce attention and the selection of particularly relevant study systems. In particular, inspiration can be found in *comparative* biology, a discipline that faces a similar challenge in being confronted with a huge diversity of organisms. Biologists cannot study, in exquisite detail, every organism, so they have to choose those organisms that are the most relevant to what they want to know. There are similar worries here as in case study choice in philosophy. Selected study systems might not be representative. A small number of model organisms dominate research. This can obscure diversity, or mislead biologists as to the nature of the living world (Leonelli & Ankeny, 2013). How can these worries be ameliorated?

Often, in biology, choice of study organism is made with a target phenomenon in mind, that is, some specific thing, such as a trait, activity, organ, process, etc. they would like to better understand. The target phenomenon may be instantiated in a wide variety of organisms, or only a few, and it may be instantiated differently in different cases. How, then, to pick a good study case for investigation? Insights can, of course, be generated by repeatedly studying the same cases, as with model organisms (Ankeny & Leonelli, 2020). But there are also benefits to studying a wide range of different and peculiar systems. That is what the Krogh principle – in the interpretation of Green et al. (2018) which we follow – recommends: that the study of a given biological phenomenon can be fruitfully approached via the study of those organisms which display it in a particularly distinctive or accessible way, such as through organisms with unique or extreme adaptations to specific contexts (Green et al., 2018, p. 1)^1^. This is an alternative to choosing organisms which display some phenomenon in a common or typical, but perhaps less apparent or less evident way.

As an example, some snakes have unique digestive systems which expand to an unusual degree upon feeding, and then contract afterwards, often to then lie unused for long periods of time. Such behaviour is not possible to this degree in many other organisms, including humans, and so is rare and not typical or obviously representative of broader modes of digestion at all. But the snake digestive system provides a distinctive case for gaining insight into metabolic regulation, and what happens to metabolic systems when large quantities of food are ingested in a short space of time, followed by long periods of scarcity (Green et al., 2018). It offers a unique gateway to identify the challenges faced by metabolic systems when pushed to the extreme.

The idea is that studying extreme or unusual adaptations such as these provides insights into the phenomenon of interest more broadly. What is gained from studying metabolism and digestion in snakes then is not, as would be claimed for traditional model organisms, a direct understanding of a digestive system which is similar, related to, or representative of many other organisms (or of humans in particular). Instead, it offers a richer understanding of process of life generally, and how the same phenomenon is manifested under different conditions (Wouters, 2007; Green et al., 2018).

In a similar way, if one is interested in how plants – including more ‘normal’ or quotidian plants – deal with drought, the Krogh principle might lead an investigator to go to the desert. In a dry context, the strategies employed by plants to limit water loss are pushed to the extreme, and so made particularly accessible to the researcher. This can then be explored as a case in its own right, but also investigated to see how informative it is for understanding how plants deal with water stress in more normal contexts. In many cases, Krogh organisms can therefore act as ‘negative models’, that is, they are interesting precisely because they show how some biological phenomenon can be very different to more commonly studied cases. This distinguishes ‘Krogh organisms’ from traditional model organisms. One key asset they have is that, by virtue of the often specific and extreme contexts they live in, they can make relationships between biological structure and function, and environmental constraints, more apparent. In snakes, the relevant environmental constraints include long periods without food, which shapes the kind of adaptations that have evolved in response, and results in an extreme solution to the problems of digestion and metabolism (Green et al., 2018).

The Krogh principle, and examples of its application, offers suggestions for choice of case study in philosophy of science. In particular, to select those study systems that illustrate the phenomenon of interest in a peculiar or an extreme way, including via the exploration of cases where environmental constraints are prominent. Doing this can produce broader insights into the nature of scientific activity. Just as with organisms, sciences operate in an environment – in a literal physical sense, as well as more abstract (e.g. social) senses – that greatly shapes and constrains many features of a given area of science.

Different scientific disciplines will face different sets of constraints. As with Krogh organisms, exploring these cases, and how the constraints and the phenomena relate, as well as the relation of these cases to other areas of science, can lead to insights into the nature of that specific area of science, but also into scientific processes more broadly, i.e. can lead to variation-sensitive generalisations of the type that many philosophers seek.

Of course, whether one works with more typical or more extreme or unusual cases, the issue of generalising remains. After finding organisms which seem to demonstrate an extreme or unusual instance of a phenomenon, the degree to which this is representative for other, maybe less extreme instances of that phenomenon must then be investigated. Krogh organisms will not necessarily be representative of the same biological phenomenon in other cases. This is a key part of the process of comparative biology, and is explicitly included as a second stage in the Krogh principle (Green et al., 2018). Unlike traditional model organisms, Krogh organisms do not come with an associated sense that they necessarily represent a broad class of organisms well. Generalisation is not done a priori, because it is not taken into account in study system selection – unlike in the case of model organisms – it is subject to investigation afterwards, something we think is again of use for a philosophical context.

This open-ended representational capacity is due to the principle’s grounding in comparative physiology, that is, the aim to produce insights by juxtaposing different relevant cases. In doing so, the principle allows to reconcile the study of biological diversity with the production of generalisations in biology. Studying gills may not provide any direct insights into how lungs operate, but it does reveal broader patterns at work in the ways in which organisms obtain oxygen from their environments (Green et al., 2018). Instead of having to decide between sensitivity to the plethora of possible biological forms (splitting) and broad generalisation about biological phenomena (lumping), the two can be combined through the Krogh principle, allowing a more elegant interplay between focusing on individual cases, and inferring from them to more general insights. Generalisations can be produced by studying variation in a biological phenomenon, exploring how extreme cases of this work, examining the degree to which they represent other instances of the same phenomenon, and comparing across these cases to explore the more general ways in which these phenomena operate. This is also a useful way to also think about case study choice in philosophy of science.

## Science, constraints, and values

The Krogh principle stresses that, in biology, variation is often related closely to the environmental constraints on the organism in question: what an organisms looks like and how it functions is often related to certain challenges faced by the organism. But what does it mean to talk about constrained, or extreme, or unusual science? Scientists too operate in environments that constrain them in both a physical sense and others (such as more metaphorically or intangibly). Some such constraints have been explored by philosophers, although not always phrased in these terms. One prominent set is cognitive constraints, related to human cognitive capacities and how they shape scientific practices such as idealisation (Potochnik, 2017). Science is a cognitive activity, so our cognitive abilities and inabilities largely shape what we can and cannot do in order to reach a scientific goal. Another prominent topic is social and epistemic constraints, such as Kuhn’s epistemic virtues, which include norms and principles held by a scientific community or an individual and which influence scientific activity (Kuhn, 1977; Galison, 1995).

In recent philosophy of science, increased attention has been paid to choices, decision-making, and the agency of scientists, often framed in terms of ‘values’ influencing scientific practice^2^ (Elliott, 2022). There is overlap with the notion of constraints here: both concepts may denote things like social, cognitive, and moral factors which shape science. However, in discourse around values in science, the focus is often on *choices* made by scientists or communities in the face of ‘underdetermination’, that is, where there is a gap between the empirical evidence and the resulting scientific characterisation of a phenomenon; or when some evidence may be used to support multiple different theories. To invoke the examples we explore below, if species are to be classified, and multiple classification schemes are available, how are they to be chosen? If changes to some ecosystem need to be characterised in positive or negative terms, and multiple options for characterising them are available, how is this to be decided? Social, cognitive, moral, or other similar factors are often invoked to fill these gaps (Douglas, 2000; Dupré, 2001; Elliott & Korf, 2024).

Values can, at the same time, also be seen as constraints: if values are taken as ‘what drives us when we make decisions’, they can play roles which are less to do with explicit decision making and more to do with the causal influence (of things which aren’t simply evidence or logic) on scientific processes (Elliott & Korf, 2024). For example, feminist philosophers and biologists have fruitfully unsettled male-centric values shaping the study of various aspects of biology, leading to improvements in the scientific knowledge subsequently generated (Longino, 2017). The label ‘values’ here can denote both explicit choices made by researchers, but also more hidden and implicit factors influencing research.

Taking inspiration from the Krogh case, we bring environmental and physical constraints into more direct dialogue with these cognitive and social ones. This follows an increase in philosophical interest in research environments (Rouse, 2016; Trappes & Leonelli, 2024)^3^. What does it mean to do science on or in environments which are difficult to access for humans, and in which we cannot easily survive, such as in the sea? At sea, normal terrestrial activities may become challenging^4^. Specific technological and epistemological means are needed for science to be possible here, whether up on shallow coral reefs, or down on the deep-sea floor. Of interest here is how different kinds of constraints interact with each other, and with human values. How do our values shape how we navigate physical constraints (and vice versa)? Here, the marine sciences offer a valuable Krogh system for exploring further the topic of values in science.

## The constraints of marine environments

There are multiple ways in which marine environments constrain human activity. Many of these are interrelated, and only some of them relevant to scientific activity, and to the topic of value-laden science. A brief survey of some of these constraints will therefore be helpful. So, in what ways are the marine sciences constrained by the oceanic environment? First, some basic issues. The object of study of the marine sciences - the ocean - is very large, covering around 70% of the Earth. Solid surfaces are important for many living things, and the bulk of the Earth’s solid surfaces are found underwater (Costello et al., 2010).

But the sea is also a liquid environment. Unlike the atmosphere, it is predominately made of water, making the volume of the ocean, i.e. all three spatial dimensions of it, of greater importance as a habitat for many organisms than in gaseous environments^5^. With an average depth of around 3700 m, the ocean represents a vast area of study for marine scientists and one where the dynamics of movement are, for humans and many other organisms, substantially different to those on land (Carr et al., 2003; Dawson & Hamner, 2008; Steinberg & Peters, 2015). For humans, the ocean can be difficult to move in, with different fluid dynamics at play than in the atmosphere, and strong currents inhibiting movement.

The physical properties of the ocean make it largely inhospitable to human life without significant technological support. We cannot breathe in the ocean. The pressure exerted by ocean water rapidly increases with depth. Every 10 m of ocean water adds the equivalent pressure of the entire atmosphere of air, and so, for example, at the average depth of the ocean (3700 m), the pressure exerted on ocean-dwellers is well over 300 times that exerted on the surface at sea level (Webb, 2023, Chap. 6). This pressure presents immense logistical challenges for humans, in terms of staying alive and managing equipment, increasing the cost of scientific activity in general, and increasingly with greater depth. Temperature is also often inclement at sea: whilst surface temperatures can be above 30 °C, this tails off quickly with depth, with the average temperature of the sea being around 4 °C, and the temperature of the deep-sea around 2 °C (Webb, 2023, Chap. 6). These temperatures are largely inhospitable to humans without the use of equipment to either mediate their physiological impact, or else allow for remote access to the environment. Again, this increases the cost of ocean science research, and reduces ease of access.

The sea also presents perceptual challenges. Light travels poorly in water. Below 1000 m of seawater there is no longer sufficient light for visual systems to operate without their own light source, and it is very unusual to be able to see further than 100 m in a given stretch of water (Lythgoe, 1988, p. 57). The majority of the living surface and volume of the planet – the deep-sea and the ocean floor – is mostly in darkness. Even where there is light, extra media are required in order to make observations possible and comprehensible^6^. Refractive effects on underwater light introduce distortions into vision not present on land (Lythgoe, 1988, p. 60). Something as simple as swimming goggles can help mitigate these effects, by placing air in front of the human eye, allowing for light to enter it in a more familiar terrestrial manner (by ensuring it enters the eye at the correct angles to allow it to be focused properly). This partially compensates for the distorted view of size and distance that aquatic media otherwise give us (Gislen et al., 2003; Land, 1987). The complexity of something as mundane as vision becomes much more obvious in the ocean.

As a result of these constraints, humans have little direct observational access to anything below diving limits, and even here there are perceptual and practical limitations. These act as strong constraints on our ability to produce knowledge about the ocean. We know, as John F. Kennedy famously said, ‘less of the oceans at our feet, where we came from, than we do of the sky above our heads.’ It is still true (at time of writing) that we have higher resolution maps of the surfaces of the Moon, Venus, and Mars than the ocean floor (Copley, 2014), and remedying this requires creativity and technological infrastructure, and hence brings substantial costs.

Technological innovation can help. Scuba diving has played a key role in understanding marine environments since being developed and refined from the 1950s onwards. It contributed heavily to improved understanding of marine life, and increased the accuracy and efficiency of the identification of new fish species (Eschmeyer et al., 2010; Witman et al., 2013). The invention of scuba diving has, for this reason, been compared with the invention of the microscope, insofar as it allows for more access to an otherwise very hard to observe world (Witman et al., 2013)^7^. Submarines, drones, and remotely operated vehicles have also extended the possibility for scientific study of marine environments (Helmreich, 2009; Lehman, 2018; Fish, 2024).

But even despite these, and other, technological innovations, there is a paucity of historical data about many marine environments, particularly further back than a few decades. Marine scientists have not as often had the luxury of observing the organisms they study in their environment in real time, and when they do, they may have to do so with compromises: obscured vision and reduced temporal and spatial scales of observation (e.g., due to time and mobility limits when scuba diving) (Webb, 2012, p. 538). The result is large gaps in what is known about organisms in aquatic environments, especially those not of commercial importance (Tyler et al., 2012). In part because of this, when marine organisms and environments are studied, they can offer surprises which are of biological and philosophical import. These all present opportunities for exploring a highly-constrained mode of science: ocean observation can be expensive, marine datasets patchy and fairly short-term, and considerations of perception, mobility, and survival are of core concerns during many scientific activities in a way they are not elsewhere.

There is a further relevant constraint: urgency. Studying the ocean is urgent, given that it is centrally involved in many aspects of global change, and marine ecosystems are threatened. Threats include, for example, widespread chemical pollution, the threat of acidification caused by excessive carbon dioxide emissions, or the increasing occurrence of oceanic heatwaves (Amaya et al., 2023). These threaten to radically alter the constitution of the ocean and our relationship to it (even before we have time to study and understand it as it is today). It is also a highly prized site for current and future resource extraction, something which may be subject to expansion and acceleration in the near future (Jouffray et al., 2020).

So marine environments present strong constraints on knowledge production. But we do not wish to claim that the ocean is a fundamentally different kind of environment, nor that marine sciences are fundamentally different modes of science. In line with the second step of the Krogh principle, these differences ought to be subject to investigation, to consider how representative such cases are of a given phenomenon. Here we take these extreme-seeming cases and use them to both refine and reinforce existing philosophical theories, but also to reflect back on their own representativeness as depictions of scientific practice. This offers a way into examining different modes of doing science, in particular exploring how environmental constraints shape scientific practice alongside social and epistemic ones.

## Case one: characterising changes to coral reefs

An excellent case for examining the influence of marine constraints on scientific activity is provided by coral reef science. Here, at least from the perspective of many scientists, one particular problem – shifting baseline syndrome – is driven by the kinds of concerns listed above, particularly a lack of access to coral reefs, and so a lack of familiarity with, and lack of data about, coral reef ecosystems^8^.

The problem is this: coral reef ecosystems change, and those changes are characterised by scientists as degradation, persistence, or improvement of the condition of the reef. These changes are characterised relative to a ‘baseline’ or normal state of the ecosystem, typically in terms of the abundance and distribution of particular organisms living on the reef. Shifting baseline syndrome - a term popularised by fisheries scientist Daniel Pauly (Pauly, 1995) - arises when observers of a marine ecosystem may assume it to be in a normal, non-degraded state, whilst other observers claim it is already degraded.

This problem – of characterising some change in a system – might seem to be very familiar to other studies on the role of values in science. For example, a classic case from the values in science literature focuses on scientists seeking to understand changes to the bodily tissues of rats exposed to some chemical substance. They classify these tissues as showing benign or malignant tumours. In some cases it is unclear if the tissues should be classified one way or another (for example due to showing features of both). In such cases, values, particularly through consideration of the downstream consequences of classifying the tissue one way or another, can fill the evidential gap, and cause the scientist to characterise the changes in one specific way (Douglas, 2000). Another example is provided by the measurement of inflation in economics. Economists need to pick which prices of which goods to include in the overall average they calculate, and they do this based on concerns about representing the changes which impact human wellbeing (Dupré, 2007). Here, social values (promoting human wellbeing) act to help select between options, and so enable changes to the economy to be characterised. A broader point here is that in many cases of characterising the state of some system in science, valuable (say, culturally or economically important) features of the study system may be included over others which are taken to be less important. There is, then, a well-established set of philosophical ideas which might seem to be relevant to characterising changes to reefs.

However, when discussing even relatively accessible shallow and warm water coral reefs, emphasis is placed not on the choices of marine scientists, but on the constraints of marine environments. Difficulty accessing marine environments produces a lack of historical data about the ecosystem, and limits the ability of observers to judge the condition of the system. The basic argument is that scientists, limited by lack of access to historical data about marine systems, take the environments at the start of their careers as benchmarks for healthy or normal ecosystem functioning, or as non-degraded ecosystems (i.e. as baselines). Such baselines typically comprise of a depiction of the prevalence and distribution of various species on a reef, with some optimal level of these in mind. It is deviation from this level that they then judge as degradation, without considering how earlier historical states might have appeared (Jackson, 2001; Pauly, 1995).

Do insights into the role of values on scientific activity still matter when choice is restricted by a lack of historical evidence? It might seem not: the lack of data available about past reef states might whittle down the evidence available to be included in scientific representations of reefs so much that choices about epistemic or other goals don’t play a part. The opportunities for producing baselines might be so limited as to involve very little choice by scientists. This might seem to be supported by meta-research on shifting baseline syndrome, which does show that the concept of shifting baselines is much more strongly associated with aquatic environments than terrestrial ones. A review of the articles employing the notion of shifting baselines found that 82% of studies referring to shifting baseline syndrome were aquatically focused (Guerrero-Gatica et al., 2019, p. 4). Pauly’s original formulation of the issue similarly focused on the size and stock of fish species, tying the concept closely to the aquatic world (Pauly, 1995). There are two relevant points here. First, this is a useful case for testing the role of physical constraints on scientific practice, given that shifting baseline syndrome seems to be an unusual feature of marine science, and is often linked directly to the physical constraints of the ocean. Second, given that the more constrained of the two environments (aquatic ones) seem to suffer more from shifting baseline syndrome, this might seem to support the idea that physical constraints here triumph over any issues of value-ladenness. Perhaps value-ladenness is only relevant in more unconstrainted contexts.

What is it about aquatic environments that causes them to be so closely associated with the notion of shifting baselines? In its traditional conception, the story of shifting baselines is one with a simple cause: a lack of historic access to marine systems means we do not have good records of how they looked when they were non-degraded (Jackson, 2001, p. 200; Pauly, 1995). The idea would then be that the constraints of marine environments – as discussed above – tend to be more extreme than many terrestrial environments, resulting in a lack of contemporary and historical data for assessing them. For a long time, it has been difficult, costly, and dangerous to reliably observe and count marine organisms. If this thesis about environmental constraint is correct, marine sciences might offer a refinement of theories around scientific characterisation of evidence, and more broadly insight into the question of how scientists grapple with both inaccessible and changing environments. Do philosophical senses of value-ladenness still apply here?

But the problem of baselining is still akin to characterising changes to bodily tissues, or constructing measures of inflation, in that it does still seem to involve choices about the normal or desirable state of an ecosystem. Coral reefs, and reefs generally, have changed over time – with or without human differences – and so depending on how far back you look into their history, they will be arranged differently. Shifts in modern coral reefs may cause them to resemble long-past ecological arrangements, such as ecosystems dominated by sponges or microbes, and yet these are often not considered desirable or normal, even if they represent a pre-human-influence state (Leinfelder et al., 2012)^9^. More broadly, reefs and other ecosystems can exist in a wide range of alternative states (Done, 1992). How are such alternative ecosystem arrangements to be adjudicated between? Nature does not come pre-packaged with baseline states (Braverman, 2020; Campbell et al., 2009; Jones, 2021; Ureta et al., 2020). Simply learning more about the history of an ecosystem, while it can be used to inform judgements about the state of the reef, is not enough on its own to produce a baseline. Baselining, for that reason, can still be seen as a scientific process that is value-laden: our values, and the value of specific ecological states, determine to some extent what we consider as an undegraded ecosystem, given a range of historical options to choose from. There is still a gap here to be filled between evidence and the way this is characterised by scientists, even if there is comparatively little evidence to work from.

One clearer way to see this is through the strong focus on fish and marine mammals in marine sciences and conservation. It is no coincidence that shifting baseline syndrome was originally formulated in terms of commercially important fish stocks, nor that marine sciences more generally tend to pay the most attention to fish (Pauly, 1995; Tyler et al., 2012). There are several interrelated reasons for this: animals which are larger, more widely distributed, and of commercial value, tend to be more well studied (Tyler et al., 2012); ‘charismatic’ organisms – often animals rather than plants, or other kingdoms – may be particularly charming, well-known, or aesthetically pleasing, and so have greater cultural value for humans and therefore are better studied (Duarte et al., 2008; Unsworth et al., 2019, p. 802). Marine sciences in general have a long-shared history with fisheries research, so it is unsurprising that commercially and culturally important fish species, or edible invertebrates, might take centre stage. Marine mammals and organisms closer to us phylogenetically may also be of greater concern for many of the public, funding bodies, and scientists themselves.

This is visible in the case of shifts of coral reefs to sponge or microbial states: even if these are historical reef states, they do not support the kinds of organisms which typically matter most to us. Species counts might match up with our understanding of past states of some reefs, and still not be considered true baselines. The phenomenon of ‘tropicalisation’ shows this explicitly. The northern boundaries of tropical coral reef systems can be found in the sea near Japan, where the sea gets too cold for reef species to survive, and coral reefs give way to kelp forest. However, as the sea warms, reef fish are able to move northwards into kelp forests and begin to eat the kelp. This then opens up space for corals to follow, eventually enabling either the production of some novel ecosystem with differences from traditional reef systems and kelp forests, or the movement of the entire reef ecosystem northwards (Vergés et al., 2014, 2019). Such processes can be characterised in many ways. They can, for example, be seen as positive, as they offer a refuge for threatened coral reef systems, and in that sense represent the restoration of some baseline state but in a different place. More negatively, they can be seen as a kind of ‘invasive ecosystem’, displacing the existing ecosystem and so representing a movement away from the existing baseline state.

Despite the strong environmental constraints, then, the value attributed to specific organisms shapes the prominence they are given in the baselining process. The baselining process is complicated by the fact that we tend to focus more on certain kinds of organism^10^. Here, existing arguments about value-ladenness are not undermined but reinforced, showing a kind of invariance to even a very different kind of context: even when there is a lack of historical data about, and experience with, some environment, choices still must be made with regards to how to characterise changes to it. It is not the case, at least here, that environmental constraints reduce the problem of choice for scientists so much so that concerns about value-ladenness are no longer relevant. This is not only of benefit to philosophy of science – by showing that philosophical theory is robust across these different contexts – but also to marine scientists, who may benefit from recognising that issues with baselines are not resolvable simply by having enough facts about the past state of the reef. (This is particularly true given that for some areas of marine science, the problem of shifting baselines is seen as one of the most important challenges they face (Braverman, 2020)). In the next case, we look at whether an even more extreme marine environment might still however offer lessons for philosophical theories of value-ladenness.

## Case two: taxonomy in the deep-sea

In deep-sea contexts, the interaction between traditional notions of value-ladenness and the pressures of the environment become more interesting. The example of deep-sea taxonomy is particularly instructive. Taxonomists strive to elaborate a clear and accessible catalogue of which organisms there actually are (Brökeland & George, 2009), often an important prerequisite to the protection of such organisms. Traditionally this involves venturing through the world to collect, name, describe and classify the organisms found.

The discipline of taxonomy is particularly rife with disagreements, both on fundamental conceptual issues, and in more concrete taxonomic debates (Conix et al., 2023, Cuypers et al., 2022). Very often, there is no agreement on how organisms should be grouped in kinds and how they should be named. These disagreements have been picked up by philosophers, because they relate to fundamental philosophical debates, for example with regard to the metaphysics of natural kinds (Bird & Tobin, 2024). Biologists and philosophers alike have engaged in heated debates with regard to ‘species concepts’, i.e. broad concepts of what species are, biologically and metaphysically. Many of these are related, again, to value-ladenness, that is, the notion that the interests of the observer shape the way they classify things, for example whether they are interested in the culinary or scientific features of a plant; or whether they are concerned about maximising the chances it survives (Dupré, 2001; Conix, 2019).

The deep sea presents an interesting testbed for these ideas. In particular, these conceptual discussions about taxonomizing may not always be representative of the constraints faced by taxonomy in practice. In the ocean there is a great ‘taxonomic backlog’, with the challenge of cataloguing life being particularly urgent. This is proven by the incredible number of species unknown to science that are found in each deep-sea sampling effort (Kaiser et al., 2022; Lejzerowicz et al., 2021). The deeper one gets, the more some of the challenges described earlier are exacerbated: cost, danger, difficulty. The deep ocean, however remote, is threatened as well, for example because of increasing investment in deep-sea mining. The ocean floor is rich in various kinds of highly valuable metallic resources, such as in the form of ‘polymetallic nodules’^11^. The exploitation of these is often presented as a ‘green alternative’ to mining on land (Miller et al., 2018). However, the exploitation of these resources, for example through dredging, is highly disruptive for the local environment, destroying the habitats of the unique biota dwelling in these ecosystems. Polymetallic nodules themselves have a unique related biota adapted to – and dependent on – them (Vanreusel et al., 2016). Despite being threatened, much of these biota, both in terms of taxonomy and in terms of the ecological structuring of their communities, remains unknown.

This is a great problem. The International Seabed Authority, which regulates the exploitation of deep-sea resources in areas outside national jurisdiction, mandates economic actors exploring deep-sea mining to map ecological characteristics of their concessions, to serve as baseline for afterwards assessing the environmental impact of their activities (Christiansen et al., 2022). The detection of different kinds of life can have significant consequences: for example the sea pangolin, an oddly-charismatic iron-shelled deep-sea snail, has become a symbol for conservation of the deep ocean. Its discovery in only three small deep-sea vents earmarked for mining (totalling around the size of two football pitches) has galvanised a movement to protect those areas, showing the stakes at play in discovery and classification in life sciences (Sigwart et al., 2019).

But the factors shaping classification go beyond just the reasons for, or conservation implications of, the classification activity. In the deep ocean, questions of cost and efficiency come to the fore. Existing procedures of collecting, preserving and describing organisms – typically with a focus on morphology – may not remain the best way to characterise deep ocean biota in light of increasing threats. Traditional taxonomy is slow, and requires specific expertise that according to many is becoming increasingly rare (Löbl et al., 2023). Collecting organismal specimens several thousands of metres below the sea level is technologically challenging, is likely to bring along several biases, and has the potential to disrupt precious ecosystems through the introduction of new species.

This has led to several alternative methods being discussed, for taxonomy in general and for deep-sea taxonomy in particular (e.g., Kaiser et al., 2022). For example, it is possible to bypass difficulties in both the collection and the processing of samples by aiming to collect environmental DNA (eDNA) rather than organisms themselves. eDNA, genetic material left by organisms and present throughout their habitat, constitutes organismal traces that can be used to characterise biotic communities^12^. A recent study has pointed out that, for what concerns the identification of species (linking specimens to species known to science), methods based on the metabarcoding of eDNA cost around $3 per taxonomic unit identified. Traditional morphological methods cost ten times as much, around $30 per taxonomic unit identified, mostly due to much higher labour costs (Le et al., 2022). These differences are likely to be higher for the description of species new to science, given the labour-intensiveness related to the description of new species.

Of course, such economic differences should be compared with the epistemic advantages and disadvantages of each method. For example, while eDNA-based methods have a distinct usefulness in capturing small, hidden or rare organisms that are often missed by traditional collecting, DNA-based biodiversity assessments are much less informative than traditional taxonomy. While metabarcoding can be informative of the presence of organisms, it does not give information on abundance, size distribution, age distribution and other important ecological aspects. These limitations are particularly important when organisms unknown to science are concerned. While DNA sequences give an idea that something is present, and perhaps vaguely where it belongs in the Tree of Life, describing species on the basis of DNA sequences alone gives little insight into what these species are, in which biological properties and evolutionary strategies these sequences represent.

In the same way, biodiversity research in the deep ocean has a long history of experimenting with image-based assessments, as video imaging is easier to obtain with the use of submarine technology than (undamaged) specimens (e.g., Durden et al., 2016). On this topic too there has been debate on the informativeness of such data, and for example on whether it is legitimate to describe new species without ‘real material’ to serve as type specimens (see Marshall & Evenhuis, 2015 for an oft discussed case). Video-based biology has the advantage of speed and lower cost, but disadvantages in informativeness and reliability. As such, it appears that methodological choices in taxonomy often relate to a difficult trade-off between speeding up (or cheapening) taxonomic processes and maximising the epistemic usefulness of taxonomic data.

These are not the typical considerations discussed in philosophical literature on taxonomy, which focuses primarily on metaphysical discussions of species concepts, rather than discussions on navigating physical and economic constraints (Ereshefsky, 2022). Often, we know so little about organisms in the deep ocean that conceptual taxonomic discussions become irrelevant: on the basis of vague video footage, or a long dead specimen, it is not relevant to discuss whether it should be classified along this or that principle. One can only merely describe what one sees and give it a name.

Many philosophical case studies related to taxonomy focus on taxonomic cases in more quotidian environments, typically on land, which, where the constraints of accessibility and cost are less, leaving more room for theoretical discussions. This is one main reason why the case of taxonomy in the (deep) ocean constitutes a useful Krogh system for the philosophical study of taxonomy and classification: accounts of scientific kinds should also work several thousands of metres below sea level, otherwise their usefulness is per definition limited. For example, if they present unaffordable costs, or slow down the process of discovery in the face of increasing extinction rate, they cannot be used in practice. Considering how taxonomy ‘works’ in the ocean can nuance philosophical discussions on factors shaping taxonomy, and make them more in touch with the practical challenges faced by taxonomists.

In the deep-sea case, marine constraints are more relevant to existing philosophical theory than the earlier shallow coral reef case. Classification is swamped by practical concerns about cost and physical access to the environment, factors which must be included in order to understand how classification works in these vast underwater environments. Both shallow and deep-sea cases allow for the testing of philosophical theories against diverse contexts, but also for reflexive examination of the generalisability of insights from those cases. In this case, existing theories about classification and values ought to be modified to factor in recognition of constraints operating to significantly limit decision-making in the more traditional sense of value-ladenness, at least in environments where cost and difficulty are high.

## Conclusion and further opportunities

So, the Krogh principle suggests that drawing on diverse cases can lead to more fruitful generalisations about the nature of scientific activity. In particular, it suggests that one avenue for doing this is via the exploration of scientific activities which are subject to physical constraints from the environment, such as those of marine science. We have shown here that philosophical theories about value-ladenness can be both reinforced and refined by drawing on marine examples. In the coral reef case, the interests of the observer play a larger role in shaping baselining practices than is commonly appreciated in discussions about baselines. Marine constraints alone do not explain shifting baseline syndrome. Conversely, in the deep-sea case, the constraints of the marine environment offer an important and underappreciated part of the picture for philosophers seeking to understand taxonomic practices. Beyond concerns with epistemic and non-epistemic values, the cost and difficulty of deep-sea classification has a great impact on the strategies used to undertake it. In both cases, physical constraints interact with existing concerns about interest-relativity, but to different degrees.

Marine systems can help with testing philosophical accounts of value-ladenness. They bring new features to the fore (physical constraints) but also show that existing theories of value-ladenness are still relevant under these constraints, even if they do not give the full picture in some cases. By drawing on diverse case studies, philosophers can produce more generalisable theories but also leave space for surprises coming from unusual areas of science. Just as with organisms, areas of science need not be directly related to one another in order to be fruitfully comparable.

### Constraints as opportunities

There are many further aspects of science and philosophy which may be better illuminated by drawing on marine examples. One example not covered here in detail is the commonly invoked *mobility* difference between land and sea. In the marine context, seemingly many organisms of interest are able to move of their own accord (in three dimensions) or with ocean currents much more readily than organisms living on land (Carr et al., 2003). Arguments about the ocean which centre around mobility differences with terrestrial environments are common: see for example ‘wet ontology’ which emphasises the fluidity of marine environments (Steinberg & Peters, 2015).

But a precise fundamental difference between these two environments, in terms of mobility across a wide range of organisms, is difficult to pin down (Dawson & Hamner, 2008). Often, comparisons operate between *surface*-dwelling creatures on land and *water-column* dwelling creatures in the sea. A more apt comparison (in terms of mobility) would be between air-column and water-column dwelling creatures: for example fish and birds, plankton and aeroplankton, or humans and benthic organisms (like crabs) (Dawson & Hamner, 2008; Steele, 1991, p. 432; Webb, 2012). Further study here could look at whether talk of increased mobility* generally* in marine environments is related to which species are most focused on when we conceive of these environments, as with the case of baselining. Greater attention to fish in the sea, and to medium-sized surface-dwelling organisms on land (such as domestic mammals as pets and livestock, or culturally important plants) causes us to focus on the differences in mobility between these organisms, rather than differences and similarities across a wide range of taxa (Carr et al., 2003). Here, the value of specific organisms shapes how environmental constraints themselves are described. So the extremeness of marine cases – for example due to the constraints on and affordances of mobility in the sea – can also be reappraised through the investigation of those cases. The Krogh principle, as with organisms, can operate in a reflective mode, that is, can be used to reappraise the differences and similarities between the cases it starts with.

We have also not discussed in much detail the perceptual constraints the ocean imposes on humans, but these likewise look to be very relevant to important philosophical topics. For example, there are long-standing philosophical debates about the nature of observation in science, particularly as it pertains to belief in the existence of entities postulated by scientists. Discussion of human perceptual systems and capacities has played a major role in such debates. In particular it is often charged that science, and philosophical concepts developed around it (such as objectivity), has been heavily shaped by vision and technologies for visualisation (Daston & Galison, 1992; Haraway, 1988, p.587). Sight has formed a key example in the expounding of positions in debates over realism and anti-realism in philosophy of science (Giere, 2006; Massimi, 2022). Much of this has centred around visual examples: do we really see things through a microscope? Can clouds of vapour allow us to see charged particles moving through them? (e.g. Baker, 2022; Toon, 2014; Van Fraassen, 1980). Both science and philosophy have been accused of being ‘vision-centric’, that is, privileging sight over other sensory systems, which is perhaps unsurprising given how central visual perception is for the lives of many (but not all) humans (Lycan, 2000; Stokes & Biggs, 2014; Barwich, 2020, pp. 3–4).

The optical and perceptual properties of the media encountered by the scientist are of relevance here. The ocean, which is relatively opaque to light, and transparent to sound, provides a further set of valuable case studies for refining and testing arguments that science and philosophy privilege the visual (Stokes & Biggs, 2014). The field of marine bioacoustics (see Montgomery & Radford, 2017), or other areas of marine sciences, look to not only provide examples of science where vision plays a lesser role, but also to show how distinctions between different sensory systems may themselves be artifacts of our perspective, the kinds of organisms we are, and the kinds of environments we inhabit^13^. There are no doubt many other interesting features of marine environments which can help draw out specific philosophical issues, to the benefit of both philosophy and science.

### Broader contributions

There is a broader opportunity here to further investigate a different kind of disunity of science than that discussed at the start of this paper: the differences within and between marine and terrestrial sciences. It is not a coincidence that we invoke marine environments here and that both Krogh and Green et al. likewise draw on aquatic-terrestrial comparisons (e.g. lungs/gills, bats/whales, deserts/rivers). These environments often shape life and the activity of living things in significant ways, leading to different solutions to the problem of living (and, we argue here, sometimes to the problem of the creation of knowledge). There are direct connections between the philosophical and biological version of the Krogh principle here: insofar as philosophers want to understand the diversity of science, and different areas of science may be shaped by their study environments and organisms, philosophers should want to similarly include a diverse array of such environments and organisms in their thinking (particularly insofar as these make for extreme or unusual areas of science).

The comparative perspective we have offered here undercuts any straightforward environmental determinism. It may turn out that the differences within marine sciences, in terms of things like practices, methods and concepts, are larger than those between terrestrial and marine sciences, as has been argued for terrestrial and marine environments (Webb, 2012). In the case of coral reef science, for example, there may be strong similarities with medical sciences (Ankeny & Leonelli, 2019), something driven by a mixture of ecological and social factors (Jones, 2024).

The Krogh principle then can be seen as part of a broader question in philosophy, history and social studies of science: which factors cause differences in how science is done? When can we expect patterns to hold across diverse areas of science (Currie, 2015, p.20)? Which factors drive the differentiation of disciplines generally? A diversity-sensitive philosophy has to grapple with multiple dimensions of variation in its object of study: marine sciences may differ from one another, and from terrestrial cases, not just because of the physical nature of the environment, but because of already-existing social and institutional factors (e.g. different journals, conferences, funding bodies), or for economic reasons (e.g. focus around distinct commercially important organisms or resources) (Steele, 1991; Underwood, 2005; Webb, 2012). There are important ways in which bits of marine science and terrestrial science might be particularly relevant to one another and yet operate separately, for example soil and sediment science, or, more surprisingly, studies on whales and trees, organisms which have similar roles in terms of carbon cycling in their respective environments (Dawson & Hamner, 2008; Webb, 2012, p. 536).

So for the discerning philosopher, comparisons between parts of marine and terrestrial sciences (which may themselves be disunified) offer opportunities for finding particularly accessible routes into studying certain topics, and for uncovering surprising connections between areas of science which might at first glance look very different^14^. There are intriguing temporal dimensions to these questions too, given that the marine sciences seem also to be undergoing transformations, such the increasing focus on understanding human-ocean relations, and so an upsurge in socially-oriented studies, aimed at better understanding how the ocean and human civilisation relate to one another (McKinley et al., 2020). These transformations offer an even greater potential range of case studies which can help elucidate particular phenomena of interest to philosophers, unpick different kinds of scientific patterns, and explore relations between research environments and the disciplines which form within and about them.

We hope to have shown here that philosophy and the sea are better off together. Various aspects of marine sciences should be of particular interest to the philosopher of science. Science done in the sea offers a wealth of case studies, especially when combined with comparative methods, and we cannot afford to ignore it. Incorporating these can help move towards the re-creation of a truly general philosophy of science anchored in the full range of human investigative practices and responsive to the challenges faced by both scientists and life generally.

## Data Availability

Not applicable.

## Footnotes

1 It is worth noting here at the outset that what counts as ‘extreme’ and ‘unusual’ is of course relative to the kinds of lifeforms we are, reflects the kinds of things we’re familiar with, and may well be influenced by our terrestrial (and cultural, human, mammalian, etc.) perspectives (for example, from a deep-sea-vent-dwelling organism’s perspective, we might be seen as the extremophiles). It is for precisely this reason that such cases provide important insights.

2 See Chang (2011) for such a call to pay attention to the agency of scientists. See Bollhagen (Forthcoming) for work on the agency of scientists which moves beyond discussion of values in science.

3 It also responds to calls such as “Hammer’s maxim” - to ‘include the measurer’ when measuring (Hammer, 2021).

4 Some things no doubt become easier, although discussion of the positive affordances of marine environments is beyond the scope of this paper. See the work of marine anthropologist Stefan Helmreich for exploration of how the sea is a place where science, amongst other activities, is difficult for humans p. XVI, pp.47–48 (Helmreich, 2009).

5 Although life in the atmosphere is poorly understood and so perhaps underappreciated. See, for instance, the concepts of aeroplankton (Smith, 2013) or ‘thunderstorm asthma’ (Kevat, 2020), which testify to a more lively atmospheric biome than might be expected, and call for reflection on the distinction between land and sea (something we return to in the conclusion).

6 The same is true of terrestrial macroscopic vision - e.g. air - although this is taken for granted until the impacts of the intervening media become more striking, as with e.g. mirages.

7 Whether vision underwater presents similar epistemic challenges for those who are inclined to see microscopic entities as not warranting belief is unclear (e.g. (Van Fraassen, 1980), but an interesting angle to explore in this debate.

8 See, for instance, an op-ed on this topic from a marine biologist and science communicator in the LA Times (Olson, 2002).

9 For more on baselines, history, and connections to the naturalistic fallacy, see Bianchi and Watkins (Forthcoming).

10 Note that shifting baseline syndrome is a problem for potentially any ecosystem undergoing change, and so the conclusions drawn here may have relevance elsewhere too. There are also connections here with the ‘reference class problem’ in philosophy of medicine (Kingma, 2007; Jones, 2021).

11 For more on these, see Brandt, 2025 (https://link.springer.com/article/10.1007/s40656-025-00682-6).

12 For more on eDNA and values, see Shea, Forthcoming (10.1007/s40656-025-00692-4).

13 For example: does sensing the movement of particles directly with hair-like appendages, rather than the sound wave with a membrane (as in humans), count as hearing, touch, or something in between? Others have pointed out that marine organisms often confound the distinctions between other senses too, such as touch and taste, or touch and sight (Hayward, 2010); or that the distinction between taste and smell are complicated by considering both terrestrial and marine examples (Mollo et al. 2022). The point here is not that marine environments produce fundamentally different sensory systems, but that studying the particular ways in which marine environments constrain both organism perception and the related practices of science offers insights into physiological, ecological and scientific processes, which can be used to enrich resulting philosophical concepts.

14 There are movements to make this kind of practice in philosophy of science more explicit: for example, the *Journal for General Philosophy of Science* had in early 2025 a call for papers for a special issue on comparative philosophy of science.
